# The influence of AI literacy on pre-service teachers’ learning motivation: the mediating role of basic psychological needs

**DOI:** 10.3389/fpsyg.2026.1831770

**Published:** 2026-05-08

**Authors:** You Wang, Kunyue Yang, Dong Han

**Affiliations:** 1 School of Foreign Languages and Cultures, Jilin University, Changchun, Jilin, China; 2 School of Information Science and Technology, Northeast Normal University, Changchun, China

**Keywords:** AI literacy, basic psychological needs, learning motivation, pre-service teachers, self-determination theory

## Abstract

**Introduction:**

The rapid integration of generative AI into higher education has raised critical questions about how learners’ intrinsic motivation is transformed. Pre-service teachers, as both current learners and future educators, represent a particularly important population for examining this issue.

**Methods:**

This study employed an explanatory sequential mixed-methods design. A sample of 600 pre-service teachers completed questionnaires assessing four dimensions of AI literacy (awareness, application, evaluation, ethics), basic psychological needs (autonomy, competence, relatedness), and learning motivation. Structural equation modeling was used to test hypothesized relationships. Subsequently, semi-structured interviews were conducted with 36 pre-service teachers and 8 course instructors to provide qualitative elaboration.

**Results:**

AI awareness and application negatively predicted autonomy (*β* = –0.190 and –0.332, respectively), while AI evaluation and ethics positively predicted competence (*β* = 0.315, 0.326) and relatedness (*β* = 0.333, 0.419). All three psychological needs mediated the relationships between AI literacy dimensions and learning motivation, with competence showing the strongest indirect effects (0.135-0.143). Contrary to predictions from social comparison and technostress theories, AI awareness positively predicted competence (*β* = 0.142) and relatedness (*β* = 0.172).

**Discussion:**

The findings reveal differentiated effects of AI literacy dimensions on basic psychological needs. An “identity-context dual boundary” framework is proposed to explain when AI awareness becomes empowering rather than threatening. These results extend self-determination theory to intelligent educational contexts and provide practical guidance for AI literacy curriculum design in teacher education.

## 1 Introduction

The rapid advancement of artificial intelligence technologies is fundamentally transforming higher education learning modalities to an unprecedented extent. Generative AI systems, exemplified by ChatGPT-4 and DeepSeek, have become increasingly integrated into university students’ knowledge construction processes, serving as ubiquitous and efficient auxiliary tools for academic inquiry. Pre-service teachers, as a population characterized by elevated technological awareness, demonstrate particularly notable frequency and depth of AI utilization (Yuan et al., 2025). However, as AI begins to assume tasks traditionally considered higher-order cognitive functions, a critical question has emerged that demands urgent attention from educational researchers in the intelligent age: How will learners’ intrinsic motivation—rooted in personal interest and value identification—be transformed?

For pre-service teachers, this question carries strategic significance transcending individual development. As both current learners and future educators, their technological literacy, learning motivation, and behavioral patterns will shape the developmental pathways of subsequent generations’ core competencies through their pedagogical practice. Since 2022, Chinese educational policies have provided systematic responses to this imperative: from the Teacher Digital Literacy framework designating digital awareness and ethics as core teacher competencies, to the Action Plan for Deepening Basic Education Curriculum and Teaching Reform emphasizing technology-education integration capabilities, and the 2024 National Education Work Conference explicitly advocating for education digitalization to guide educational modernization—cultivating teaching staff with advanced AI literacy has become an urgent practical necessity (Ministry of Education of the People's Republic of China, 2022, 2024). These policy directives not only guide pre-service teacher preparation but also necessitate theoretical reconceptualization of traditional learning motivation mechanisms: when AI becomes deeply embedded in learning processes, how is intrinsic motivation—originating from personal interest—generated and sustained, a question that requires fresh theoretical perspectives for adequate analysis?

From a learning psychology perspective, AI technology exerts dual effects on learning motivation. On one hand, AI can provide immediate feedback, personalized support, and cognitive scaffolding, thereby enhancing learners’ sense of autonomy and competence and stimulating intrinsic motivation (Son et al., 2025). On the other hand, technological convenience may induce cognitive offloading and intellectual inertia, undermining deep learning engagement and learner agency (Watts, 2025; Romeo and Conti, 2025). This suggests that the relationship between AI literacy and learning motivation is not simply linear and facilitative; rather, its pathways of influence may vary depending on individuals’ satisfaction of basic psychological needs. Nevertheless, current AI literacy education in teacher training institutions predominantly emphasizes technical skill acquisition, with insufficient systematic attention to the psychological effects and motivational dynamics accompanying AI use. Responding to both the developmental imperatives of the intelligent age and national educational policy directions, the present investigation systematically examines the intrinsic associations between pre-service teachers’ AI literacy and learning motivation, along with the psychological mechanisms transmitting these effects. This endeavor both tests and extends self-determination theory (SDT) within intelligent educational contexts while providing empirical foundations for optimizing pre-service teacher preparation systems.

### 1.1 AI literacy: conceptualization and dimensional structure

As a critical extension of core competencies in the intelligent age, the conceptualization and dimensional delineation of AI literacy constitute the theoretical foundation for understanding how technology shapes learners. The construct of “competence” originates from the concurrent development of psychology and linguistics in the mid-twentieth century (White, 1959; Chomsky, 1965), with its widespread influence on international education policy beginning with the OECD’s “Definition and Selection of Key Competencies” project (OECD, 2003).

With the large-scale educational integration of artificial intelligence, AI literacy has emerged as a major research focus. Long and Magerko (2020) proposed an influential definition characterized by three core elements: critical examination of AI systems, human-AI collaboration, and contextual application. At the policy level, UNESCO (2024) released the AI Competency Framework for Students, delineating core dimensions including human-centered mindset, AI ethics, AI techniques and applications, and AI system design. In academic research, Ng et al. (2021) synthesized multidisciplinary literature to propose a four-dimensional framework comprising AI Awareness, AI Application, AI ethics, and AI Evaluation—a conceptualization aligned with Long and Magerko's (2020) definition.

Recent studies have further validated this framework’s applicability. Ayanwale et al. (2024) empirically investigated Nigerian pre-service teachers and found that AI ethics and attitudes critically influence technology integration intentions, confirming the cross-cultural applicability of the four-dimensional structure. Funa and Gabay (2025) examined pre-service teachers preparing to teach “Generation Alpha” and identified that ChatGPT literacy encompasses technical proficiency, cognitive criticality, and ethical awareness, with cognitive criticality demonstrating the strongest predictive effect on teaching integration intentions. Solyst et al. (2025) extended the framework from a critical AI literacy perspective, emphasizing the need to examine power structures and social biases embedded within AI systems.

Based on pre-service teachers’ identity characteristics and learning contexts, the present study adopts Ng et al.'s (2021) four-dimensional structure—awareness, application, evaluation, and ethics—as the core analytical framework. Although Ng et al. (2021) originally labeled these dimensions as Awareness, Application, Evaluation, and Ethics (without an “AI” prefix), their conceptual definitions align closely with the present framework: Awareness corresponds to awareness of AI’s capabilities and limitations; Application reflects the ability to apply AI tools in practical tasks; Evaluation involves the critical evaluation of AI’s outputs and societal implications; and Ethics encompasses both ethical understanding and behavior.

This conceptual mapping ensures continuity with prior frameworks while employing terminology better suited to the learning context of pre-service teachers.

### 1.2 Differential effects of AI literacy dimensions

Subsequent research has revealed that different dimensions of AI literacy exert significantly differentiated—and even opposing—effects on learning psychology. Regarding awareness and application, the Cognitive Miser Principle (Fiske and Taylor, 1991) posits that humans possess a natural tendency to minimize cognitive effort. AI provides precisely such convenient cognitive alternatives, potentially inducing cognitive offloading and diminished deep learning. Automation bias theory (Parasuraman and Manzey, 2010) further demonstrates that individuals tend toward excessive trust in and reliance on automated systems’ judgments. Romeo and Conti (2025), in their systematic review, identified active user engagement as the most effective intervention for reducing automation bias and maintaining critical thinking. This suggests that AI application ability does not necessarily translate into autonomous learning; rather, it may induce cognitive dependency when learners engage passively. Watts (2025) further proposed the concept of “cognitive debt,” arguing that excessive reliance on AI to complete higher-order cognitive tasks undermines learners’ autonomous thinking capabilities.

In contrast, evaluation and ethics exhibit positive facilitative effects. From the perspective of human-AI collaborative cognition, when learners identify and correct deviations in AI outputs, their professional judgment receives immediate validation—a critical source of competence satisfaction (Son et al., 2025). From the perspective of social identity theory (Tajfel and Turner, 1986), learners adhering to AI ethical norms are more likely to receive positive social recognition from instructors and peers, thereby strengthening professional identity and group belonging. Bećirović et al. (2025) found that critical evaluation ability regarding AI outputs demonstrates the strongest predictive effect on self-efficacy. Solyst et al. (2025) noted that when learners critically examine the ethical limitations of AI systems, their professional identity is reinforced through communal deliberation. Yi and Siquan (2025) confirmed that satisfaction of basic psychological needs, mediated by AI literacy and self-efficacy, significantly influences pre-service teachers’ behavioral intentions to use AI. The collaborative evaluation, negotiation, and revision of AI outputs constitute knowledge co-construction practices that enhance cohesion and interpersonal connection within learning communities (Chiu et al., 2023).

Notably, the directional influence of AI awareness on psychological needs may not be unidimensional. On one hand, according to social comparison theory (Festinger, 1954), when pre-service teachers possess clear awareness of AI’s powerful capabilities, they may engage in “upward comparison” with AI, thereby inducing ability anxiety and competence crises (Brougham and Haar, 2018). Technostress theory (Tarafdar et al., 2007) further suggests that awareness of technological complexity itself can constitute psychological burden, while excessive immersion in human-computer interaction may weaken interpersonal connections (Turkle, 2015). On the other hand, recent research offers a different perspective: Tulis et al. (2025) found that challenge appraisal—the perception of AI as an opportunity for growth rather than a threat—positively predicts AI usage intentions through the mediating roles of autonomy and competence. This finding suggests that when individuals perceive AI capabilities as “learnable professional tools” rather than “threats to personal ability,” upward comparison may instead stimulate professional development motivation. Particularly among pre-service teachers—a population of “prospective educators”—cognitions regarding AI technology are inherently associated with future professional capacity building, potentially enabling a value shift from “tool cognition” to “professional competence cognition.” This implies that the relationship between AI awareness and psychological needs is not unidirectional; rather, its directional influence requires comprehensive judgment incorporating population characteristics and contextual conditions, with collective learning scenarios serving as important moderating variables.

### 1.3 Self-determination theory and basic psychological needs

Self-determination theory (SDT), proposed by Deci and Ryan (1985), represents one of the most influential frameworks for explaining the emergence and internalization of human motivation. The theory’s central proposition holds that satisfaction of three basic psychological needs—autonomy (the experience of volition and self-endorsement), competence (the sense of effectiveness and mastery), and relatedness (the feeling of connection and belonging)—constitutes the necessary precondition for initiating intrinsic motivation and facilitating high-quality internalization of extrinsic motivation (Ryan and Deci, 2020).

Application of SDT in educational contexts has accumulated substantial empirical support. Van den Broeck et al.'s (2016) meta-analysis confirmed that satisfaction of the three psychological needs demonstrates robust positive associations with intrinsic motivation, learning engagement, and academic achievement. In recent years, the theory has been increasingly applied to AI educational contexts. Huang and Zhao (2025) empirically investigated 511 university faculty members and found that AI literacy enhances job satisfaction and work-life balance by satisfying needs for autonomy, competence, and relatedness; technology acceptance further moderates the relationship between AI literacy and psychological need satisfaction. Tulis et al. (2025), drawing on a sample of 3,094 Austrian university students, demonstrated that challenge appraisal—the perception of AI as an opportunity for growth rather than a threat—significantly and positively predicts AI usage intentions through the mediating roles of autonomy and competence. Wut et al. (2025) found that school support promotes teachers’ AI literacy development through enhanced satisfaction of basic psychological needs, thereby revealing the critical mediating role of psychological needs in technological literacy formation.

### 1.4 Learning motivation: conceptual foundations and AI-mediated evolution

Learning motivation refers to the intrinsic drive directed toward learning activities themselves, characterized by pleasure and satisfaction derived from the learning process rather than pursuit of external rewards. Bruner (1960) emphasized that intrinsic motivation—manifested as natural tendencies toward curiosity, exploration, and knowledge-seeking—constitutes the core driver of cognitive development. Gottfried (1985) defined learning motivation as the intrinsic drive derived from pleasure and satisfaction inherent in learning itself, with the defining characteristic that the activity itself serves as the reward rather than serving external outcomes. This conceptualization aligns closely with Deci and Ryan's (1985) notion of intrinsic motivation within SDT.

In contexts characterized by deep AI integration, the mechanisms underlying the generation and evolution of learning motivation have emerged as a research focus. Bećirović et al. (2025) recently found that students’ AI literacy significantly influences their AI output quality, self-efficacy, and academic performance. Although self-efficacy is conceptually distinct from the competence dimension of basic psychological needs in self-determination theory, both constructs reflect learners’ sense of effectiveness and mastery. This finding suggests that AI literacy may enhance learning engagement through mechanisms related to competence satisfaction. Bewersdorff et al. (2025) further distinguished the differential pathways through which AI Evaluation and AI literacy influence AI self-efficacy. They found that AI literacy indirectly enhances learning engagement by strengthening individuals’ sense of control over technology, whereas AI Evaluation more directly affect willingness and frequency of technology use. Empirical research targeting pre-service teachers has similarly demonstrated that AI literacy influences behavioral intentions to use technology through the mediating role of self-efficacy (Yi and Siquan, 2025). This finding aligns with Funa and Gabay's (2025) research, suggesting that in an era of deep AI integration, learning motivation stems not only from instrumental competence but also from critical mastery of technology.

### 1.5 Research gaps and the present study

Synthesizing the extant literature, different dimensions of AI literacy exhibit differentiated pathways of influence on basic psychological needs. Regarding awareness and application, research has revealed negative psychological mechanisms including cognitive offloading and the technology convenience paradox (Watts, 2025; Romeo and Conti, 2025): heightened AI awareness may reduce willingness to engage in active thinking, while advanced AI application ability may undermine the sense of autonomous control. Regarding evaluation and ethics, research supports positive effects: collaborative evaluation, negotiation, and revision of AI outputs constitute important knowledge co-construction practices that enhance cohesion and interpersonal relationships within learning communities (Chiu et al., 2023; Solyst et al., 2025).

Based on the foregoing synthesis, the present study identifies three critical research gaps. First, most studies conceptualize AI literacy as a unitary construct, with insufficient attention to the differentiated effects of its four-dimensional structure. Second, specialized research targeting pre-service teachers as a dual-identity population remains underdeveloped. Third, the mediating mechanisms of basic psychological needs in the relationship between AI literacy and learning motivation have not been adequately examined. This study addresses these gaps through an explanatory sequential mixed-methods design, systematically examining how the four dimensions of AI literacy differentially influence basic psychological needs and, subsequently, learning motivation. The specific research hypotheses are developed below.

### 1.6 Research hypotheses

Building on the foregoing theoretical synthesis and employing self-determination theory as the overarching framework, this study integrates the differentiated effects of the four-dimensional structure of AI literacy to propose the following hypotheses.

#### 1.6.1 AI awareness and AI application: technology dependence risks

The Cognitive Miser Principle (Fiske and Taylor, 1991) and automation bias theory (Parasuraman and Manzey, 2010) indicate that humans possess a natural tendency to minimize cognitive effort and are prone to excessive trust in automated systems. Heightened AI awareness may induce cognitive offloading, while advanced AI application ability may shift the learning process from “human-led” to “technology-led,” thereby undermining the sense of autonomous control (Watts, 2025; Romeo and Conti, 2025). Accordingly, we propose:

*H1a*: AI awareness significantly negatively predicts pre-service teachers' autonomy.

*H2*: AI application significantly negatively predicts pre-service teachers' autonomy.

From the perspective of social comparison theory (Festinger, 1954), clear awareness of AI’s capabilities may trigger “upward comparison,” thereby inducing ability anxiety and competence crises (Brougham and Haar, 2018). Technostress theory (Tarafdar et al., 2007) also indicates that awareness of technological complexity itself can constitute psychological burden, while excessive immersion in human-computer interaction may weaken interpersonal connections (Turkle, 2015). Based on these theoretical perspectives, AI awareness might negatively predict competence and relatedness.

However, recent research offers an alternative theoretical expectation: when individuals perceive AI as an opportunity for growth rather than a threat (challenge appraisal), AI awareness may positively predict psychological need satisfaction through pathways of autonomy support and competence confirmation (Tulis et al., 2025; Son et al., 2025). Given that pre-service teachers possess a dual identity as both learners and future educators, their cognitions regarding AI may be inherently associated with professional competence construction. Whether the negative effects predicted by traditional technology threat theories hold within this population remains an empirical question. Accordingly, we propose:

*H1b*: AI awareness significantly predicts pre-service teachers' competence (direction to be tested).

*H1c*: AI awareness significantly predicts pre-service teachers' relatedness (direction to be tested).

It is worth noting that H1a and H1b/H1c target different psychological needs with distinct theoretical rationales. The hypothesized negative effect of AI awareness on autonomy (H1a) is grounded in the Cognitive Miser Principle and automation bias theory, which describe universal cognitive tendencies toward effort minimization and system over-reliance. These mechanisms are relatively robust across populations and may be less directly susceptible to contextual moderation compared to the identity-related processes underlying competence and relatedness. In contrast, the effects of AI awareness on competence and relatedness (H1b, H1c) involve more socially and identity-laden processes—namely, how individuals interpret technological capabilities in relation to their self-concept and professional aspirations. As such, these effects may be more sensitive to population characteristics (e.g., pre-service teachers’ dual identity as learners and future educators) and learning contexts (e.g., collaborative vs. individual use). Therefore, while H1a is specified with a clear directional prediction, H1b and H1c are formulated as exploratory hypotheses with direction to be determined empirically.

It is worth noting that H1b and H1c are formulated as exploratory hypotheses. Rather than making directional predictions, they are designed to test competing theoretical perspectives—technological threat theories (which would predict negative effects) versus professional identity theory (which would predict positive effects). The empirical results will indicate which theoretical framework better explains pre-service teachers’ psychological responses to AI awareness.

#### 1.6.2 AI evaluation and AI ethics: cognitive mastery effects

When learners can critically examine, validate, and correct AI outputs, their professional judgment receives immediate validation through authentic tasks—a critical source of competence satisfaction (Deci and Ryan, 1985; Son et al., 2025). Students capable of effectively evaluating AI outputs demonstrate significantly higher self-efficacy (Bećirović et al., 2025). Funa and Gabay's (2025) research on pre-service teachers further revealed that critical evaluation ability constitutes the key to transforming technological literacy into pedagogical motivation—a finding the present study extends to the domain of basic psychological needs. The collaborative evaluation and negotiation of AI outputs constitute knowledge co-construction social interactions that enhance peer connectedness (Chiu et al., 2023). From the perspective of social identity theory (Tajfel and Turner, 1986), learners who adhere to AI ethical norms are more likely to receive positive social recognition, thereby strengthening professional identity and group belonging (Yi and Siquan, 2025). Based on these considerations, we propose:

*H3*: AI evaluation significantly positively predicts pre-service teachers' competence.

*H4*: AI evaluation significantly positively predicts pre-service teachers' relatedness.

*H5*: AI ethics significantly positively predicts pre-service teachers' competence.

*H6*: AI ethics significantly positively predicts pre-service teachers' relatedness.

#### 1.6.3 The mediating role of basic psychological needs

The core proposition of self-determination theory states that environmental factors indirectly influence individuals’ intrinsic motivation by either satisfying or frustrating the three basic psychological needs of autonomy, competence, and relatedness (Deci and Ryan, 1985; Ryan and Deci, 2020). In educational contexts characterized by deep AI integration, AI literacy constitutes an important environmental factor whose influence on learning motivation follows this same mechanism (Huang and Zhao, 2025; Tulis et al., 2025). Network analysis has further revealed that competence satisfaction surpasses autonomy and relatedness in importance within AI learning contexts, emerging as the central node connecting the motivational system. Based on these theoretical and empirical foundations, we propose:

*H7a*: Autonomy serves as a parallel mediator between AI awareness, AI application, and learning motivation.

*H7b*: Competence serves as a parallel mediator between AI evaluation, AI ethics, and learning motivation.

*H7c*: Relatedness serves as a parallel mediator between AI evaluation, AI ethics, and learning motivation.

**Figure 1:**
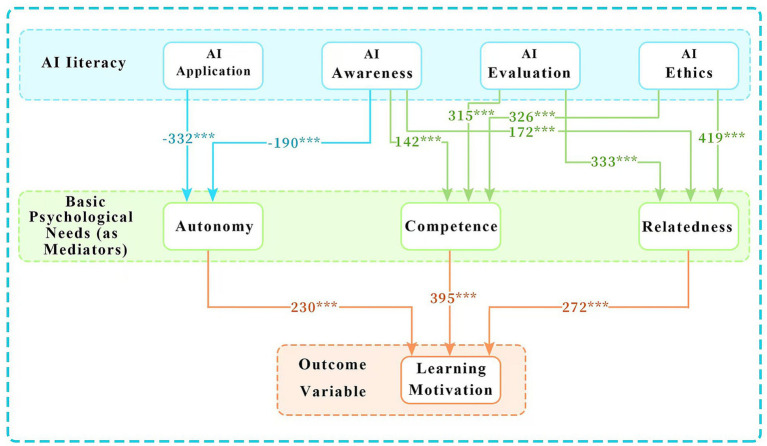
Theoretical model of the influence of AI literacy on learning motivation.

The proposed theoretical model is illustrated in Figure 1.

## 2 Materials and methods

### 2.1 Research design

This study employed an explanatory sequential mixed-methods design. In the first phase, we conducted to examine the influence of the four-dimensional structure of AI literacy on learning motivation and the mediating role of basic psychological needs (*N* = 600). In the second phase, informed by stratified quantitative results, semi-structured interviews were conducted with 36 pre-service teachers and 8 course instructors, enabling triangulation of student experiences and instructor observations. This approach constituted an integrated research design in which quantitative methods test the structure, while qualitative methods elucidate the mechanisms.

### 2.2 Participants

Quantitative Phase. The formal survey was conducted from September to October 2025 with undergraduate pre-service teachers at Northeast Normal University. A combination of stratified convenience sampling and snowball sampling was employed. Questionnaires were distributed online via Wenjuanxing (a Chinese online survey platform). A total of 650 questionnaires were distributed. After screening via reverse-item logic checks and patterned response examination, 600 valid responses were obtained, yielding an effective response rate of 92.31%. Sample demographic characteristics are presented in Table 1.

**Table 1:** Demographic characteristics of the sample (*N* = 600).

Variable	Category	Frequency (n)	Percentage (%)
Gender	Male	257	42.8
	Female	343	57.2
Grade level	First year	163	27.2
	Second year	156	26.0
	Third year	146	24.3
	Fourth year	135	22.5
Academic discipline	Humanities and social sciences	170	28.3
	Mathematics and natural sciences	168	28.0
	Arts and physical education	135	22.5
	Other	127	21.2

Percentages may not sum to 100 due to rounding. The “Other” category in academic discipline (21.2%) includes students from vocational teacher training programs (e.g., early childhood education, special education), interdisciplinary programs (e.g., educational technology, STEM education), and non-traditional teaching tracks (e.g., second bachelor’s degree programs in education, continuing education programs for teacher certification).

*Qualitative Phase*. Based on quantitative survey results, purposive sampling was employed to select 36 pre-service teachers and 8 course instructors for semi-structured interviews. The student sample was stratified according to AI literacy levels and learning motivation scores: the 600 valid responses were ranked by total scores, and 12 students were drawn from each of the high-scoring group (top 27%), middle-scoring group (middle 46%), and low-scoring group (bottom 27%), ensuring coverage across all three levels. (While a 2 × 2 stratified design (high/low AI literacy × high/low learning motivation) would have been more rigorous, the total-score approach was chosen to ensure sufficient group sizes. See Section 4.6 for discussion of this limitation.) The instructor sample included faculty teaching courses in education, educational technology, and subject-specific pedagogy.

The 8 course instructors who participated in the qualitative interviews included 5 males and 3 females, with ages ranging from 32 to 51 years (M = 41.2, SD = 6.7). Their teaching experience ranged from 8 to 25 years (M = 15.4, SD = 5.2). Regarding AI knowledge, 2 instructors self-reported ‘advanced’ proficiency, 4 reported ‘intermediate,’ and 2 reported ‘beginner’ level. All instructors had integrated AI tools into their teaching to varying degrees. This demographic information is relevant because instructors’ prior experience with AI may influence their observational perspectives on students’ AI literacy and learning motivation.

### 2.3 Measures

The questionnaire was developed with self-determination theory (Deci and Ryan, 1985) as the core framework, integrating and adapting items based on established scales. All items were measured on a 5-point Likert scale (1 = strongly disagree, 5 = strongly agree), with reverse-scored items transformed using “6 minus the original score” to ensure consistent directional scoring. The complete questionnaire consisted of five sections: demographic variables, AI usage behaviors, AI literacy scale, basic psychological needs scale, and learning motivation scale. All scales are presented in full in Appendix A.

#### 2.3.1 AI literacy

The AI literacy scale was developed based on Ng et al.'s (2021) four-dimensional framework, encompassing awareness, application, evaluation, and ethics, with four items per dimension, totaling 16 items. Although Ng et al. (2021) originally labeled these dimensions as Awareness, Application, Evaluation, and Ethics, their conceptual definitions align closely with the dimensions adopted in this study: Awareness corresponds to awareness of AI’s capabilities and limitations; Application reflects the ability to apply AI tools in practical tasks; Evaluation involves the critical evaluation of AI’s outputs and societal implications; and Ethics encompasses both ethical understanding and behavior.

#### 2.3.2 Basic psychological needs

The basic psychological needs scale encompassed three dimensions—autonomy, competence, and relatedness—with four items per dimension, totaling 12 items, adapted from Van den Broeck et al.'s (2010) work context scale with wording modified to suit the learning context. Autonomy refers to the experience of volition and self-endorsement in learning activities; competence reflects the sense of effectiveness and mastery; and relatedness captures the feeling of connection and belonging with peers and instructors.

#### 2.3.3 Learning motivation

The learning motivation scale was developed based on Gottfried (1985) conceptualization of intrinsic motivation, comprising four items assessing the extent to which participants engaged in learning activities for the inherent pleasure and satisfaction derived from the learning process itself.

#### 2.3.4 Pilot testing

To examine the scientific rigor and applicability of the questionnaire, a pilot test was conducted from April to May 2025 with undergraduate pre-service teachers at Northeast Normal University. Convenience and snowball sampling were employed, and questionnaires were distributed via Wenjuanxing. A total of 320 questionnaires were collected. After screening via reverse-item logic checks and patterned response examination, 304 valid questionnaires were obtained, yielding an effective response rate of 95.00%. The pilot sample represented all grade levels and academic disciplines.

Based on the pilot results, an AI usage behavior module was added (encompassing usage frequency, usage scenarios, and usage purposes, totaling four items), and minor wording adjustments were made to individual items to enhance clarity, resulting in the final questionnaire used in the main study. Detailed pilot results are reported in Section 3.1.

**Figure 2:**
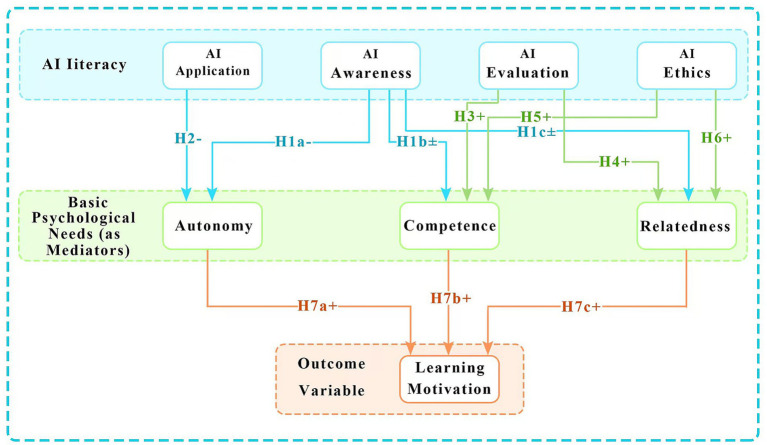
Structural equation modeling results for the influence of AI literacy on learning motivation.

The AI usage behavior module assessed the frequency, purpose, and context of AI tool use. The composite score from this module is labeled ‘Objective AI Use’ in Table 2. This module was designed for descriptive purposes only and was not included in the main structural equation model (Figure 2; Table 3) because the theoretical focus of this study was on AI literacy as a multidimensional competency (awareness, application, evaluation, ethics) rather than on behavioral frequency measures. The correlations between objective AI use and other variables are reported in Table 2 for exploratory purposes.

**Table 2:** Descriptive statistics and correlations among study variables (*N* = 600).

Dimension	M	SD	1	2	3	4	5	6	7	8	9
AI awareness	3.00	1.14	1								
AI application	2.99	1.16	0.28**	1							
AI evaluation	2.97	1.08	0.19**	0.23**	1						
AI ethics	3.04	1.17	0.24**	0.24**	0.32**	1					
Autonomy	3.03	1.18	−0.26**	−0.34**	−0.049	−0.047	1				
Competence	3.01	1.14	0.14**	0.16**	0.35**	0.37**	−0.04	1			
Relatedness	2.97	1.17	0.17**	0.22**	0.41**	0.46**	−0.05	0.32**	1		
Learning motivation	2.97	1.12	0.037	0.014	0.23**	0.23**	0.16**	0.40**	0.34**	1	
Objective AI use	2.53	0.51	0.11**	0.09*	0.21**	0.13**	0.01	0.18**	0.15**	0.27**	1

M, mean; SD, standard deviation. Diagonal entries (—) represent the correlation of each variable with itself. ** *p* < 0.05; *p < 0.01 (two-tailed).

**Table 3:** Path coefficients from structural equation modeling (*N* = 600).

Hypothesis	Path	Standardized β	Unstandardized β	S.E.	C.R.	*p*	Result
H1a	AI awareness → autonomy	−0.190	−0.208	0.055	−3.804	<0.001	Supported
H1b	AI awareness → competence	0.142	0.156	0.055	2.836	0.005	Supported (exploratory, positive association found)
H1c	AI awareness → relatedness	0.172	0.189	0.055	3.436	<0.001	Supported (exploratory, positive association found)
H2	Application → autonomy	−0.332	−0.343	0.053	−6.438	<0.001	Supported
H3	AI evaluation → competence	0.315	0.347	0.060	5.733	<0.001	Supported
H4	AI evaluation → relatedness	0.333	0.367	0.058	6.367	<0.001	Supported
H5	AI ethics → competence	0.326	0.328	0.054	6.125	<0.001	Supported
H6	AI ethics → relatedness	0.419	0.423	0.053	8.000	<0.001	Supported
H7a	Autonomy → learning motivation	0.230	0.232	0.045	5.106	<0.001	Supported
H7b	Competence → learning motivation	0.395	0.413	0.054	7.645	<0.001	Supported
H7c	Relatedness → Learning Motivation	0.272	0.283	0.051	5.540	<0.001	Supported

Model fit indices: CFI = 0.984, TLI = 0.982, RMSEA = 0.022, SRMR = 0.068, indicating excellent model fit. S.E. = standard error; C.R. = critical ratio.

### 2.4 Qualitative procedure

The interview protocol was structured around core themes: AI usage experiences, changes in psychological needs, evolution of learning motivation, and instructor observational perspectives. Each interview lasted 30–60 min and was conducted in a private setting on campus. All interviews were audio-recorded with participants’ consent and transcribed verbatim, yielding approximately 35 h of audio recordings and 250,000 words of transcribed text.

### 2.5 Data analysis

#### 2.5.1 Quantitative analysis

Quantitative data were analyzed using SPSS 26.0 and AMOS 24.0. The following analyses were conducted:

First, common method bias was assessed using Harman’s single-factor test via unrotated exploratory factor analysis on all core items. Second, descriptive statistics (means, standard deviations, skewness, kurtosis) and internal consistency reliability (Cronbach’s *α*) were calculated for each dimension. Third, Pearson correlation analyses were conducted to examine bivariate relationships among all study variables. Fourth, confirmatory factor analysis (CFA) was performed to evaluate discriminant validity among the eight core constructs (AI awareness, AI application, AI evaluation, AI ethics, autonomy, competence, relatedness, and learning motivation). Model fit was assessed using multiple indices: χ^2^/df, comparative fit index (CFI), Tucker–Lewis index (TLI), root mean square error of approximation (RMSEA), and standardized root mean square residual (SRMR), with recommended thresholds of CFI/TLI ≥ 0.90, RMSEA ≤ 0.08, and SRMR ≤ 0.08 indicating acceptable fit (Hu and Bentler, 1999).

Fifth, structural equation modeling (SEM) was employed to test the hypothesized relationships (H1a through H7c). Sixth, bias-corrected bootstrap analysis with 5,000 resamples was used to test the mediating effects of basic psychological needs, with 95% confidence intervals excluding zero indicating significant mediation.

#### 2.5.2 Qualitative analysis

Qualitative data were analyzed using thematic analysis, following the six-phase framework proposed by Braun and Clarke (2006): (1) familiarization with data through repeated reading of transcripts; (2) generating initial codes; (3) searching for themes; (4) reviewing themes; (5) defining and naming themes; and (6) producing the final report. The coding process was managed using NVivo 12 software.

This study employed qualitative triangulation strategies to enhance analytical trustworthiness. First, data source triangulation was achieved by collecting interview data from both students and instructors, enabling cross-validation from the perspectives of technology users and external observers. Second, researcher triangulation was employed: two researchers independently coded interview transcripts, with discrepancies resolved through discussion. Third, between-methods triangulation was achieved by interpreting qualitative findings in conjunction with quantitative path analysis results, forming a bidirectionally validated integration.

## 3 Results

### 3.1 Pilot study results

A pilot test was conducted with 304 pre-service teachers to examine the reliability and validity of the instruments prior to the main study. Harman’s single-factor test indicated that the first factor explained 37.75% of the variance, below the 40% threshold (Podsakoff et al., 2003), suggesting no severe common method bias. The Cronbach’s *α* coefficient for the complete questionnaire was 0.94, with dimension-specific α coefficients ranging from 0.82 to 0.89, indicating good reliability. The KMO value was 0.934, and Bartlett’s test of sphericity was significant (χ^2^ = 5869.11, df = 496, *p* < 0.001), confirming the data’s suitability for factor analysis. Exploratory factor analysis extracted eight factors with eigenvalues greater than 1, corresponding closely to the theoretical dimensions, and the cumulative variance explained was 73.11%. Item factor loadings ranged from 0.678 to 0.811, indicating good construct validity. Detailed reliability and factor analysis results for each dimension are presented in Table 4.

**Table 4:** Reliability and exploratory factor analysis results for pilot test dimensions (*N* = 304).

Core variable	Dimension	Items	Cronbach’s *α*	Factor loading range	Variance explained after rotation (%)
AI Literacy	Awareness	4	0.88	0.736~0.781	9.50%
	Application	4	0.85	0.725~0.804	9.43%
	Evaluation	4	0.82	0.678~0.773	9.37%
	Ethics	4	0.86	0.722~0.730	9.28%
Basic psychological needs	Autonomy	4	0.86	0.730~0.811	9.17%
	Competence	4	0.88	0.741~0.786	9.12%
	Relatedness	4	0.86	0.735~0.768	9.01%
Learning motivation	Motivation	4	0.89	0.735~0.768	8.13%
Complete questionnaire		32	0.94	0.678~0.811	73.11%

KMO = 0.934; Bartlett’s test of sphericity: χ^2^ = 5869.11, df = 496, p < 0.001; *N* = 304 (pilot sample).

Preliminary correlational analyses from the pilot study provided initial support for the core hypotheses. Awareness and application demonstrated significant negative correlations with autonomy (r = −0.21, −0.17, respectively, *p* < 0.01), preliminarily confirming the expectation that technological dependence may undermine autonomy. Evaluation and ethics exhibited moderate positive correlations with both competence and relatedness (r = 0.43–0.52, *p* < 0.01), consistent with the hypothesized positive effects of critical mastery on psychological needs. The three basic psychological needs also showed significant positive correlations with learning motivation (r = 0.38–0.56, *p* < 0.01). The absolute values of all inter-variable correlations were below 0.70, indicating no severe multicollinearity concerns.

Notably, pilot data revealed that AI awareness demonstrated moderate positive correlations with both competence (r = 0.538, *p* < 0.01) and relatedness (r = 0.539, *p* < 0.01). These findings, which appeared inconsistent with the negative relationships hypothesized in H1b and H1c, awaited further examination in the formal study.

### 3.2 Common method bias assessment

Harman’s single-factor test was conducted using unrotated exploratory factor analysis on all core items from the formal survey. Results indicated that the first factor explained 21.44% of the total variance, substantially below the 40% threshold, suggesting that common method bias did not pose a serious threat to the validity of the findings and that the data were suitable for subsequent analyses.

### 3.3 Descriptive statistics and reliability analysis

Means, standard deviations, skewness, kurtosis, and reliability coefficients for each dimension in the formal sample are presented in Table 5. Absolute skewness values for all dimensions were below 0.10, and absolute kurtosis values were below 1.20, indicating that the data were normally distributed and suitable for parametric analyses. Reliability analysis demonstrated that Cronbach’s *α* coefficients ranged from 0.78 to 0.85 across dimensions. Except for AI evaluation (α = 0.78), all dimensions achieved coefficients above 0.80, indicating good internal consistency reliability.

**Table 5:** Descriptive statistics and reliability analysis by dimension (*N* = 600).

Dimension	Items	M	SD	Skewness	Kurtosis	Cronbach’s α
AI Awareness	4	3.00	1.14	0.00	1.01	0.83
AI Application	4	2.99	1.16	0.01	1.08	0.84
AI Evaluation	4	2.97	1.08	0.07	0.92	0.78
AI Ethics	4	3.04	1.17	0.07	1.10	0.83
Autonomy	4	3.03	1.18	0.03	1.16	0.85
Competence	4	3.01	1.14	0.03	0.99	0.82
Relatedness	4	2.97	1.17	0.01	1.04	0.84
Learning motivation	4	2.97	1.12	0.09	1.05	0.80

M, mean; SD, standard deviation. All items measured on 5-point Likert scales.

### 3.4 Correlational analyses

Pearson correlation analyses were conducted to examine relationships among all study variables. Means, standard deviations, and the correlation matrix are presented in Table 2.

### 3.5 Confirmatory factor analysis

To examine discriminant validity among the core variables, confirmatory factor analyses were conducted comparing an eight-factor model, a seven-factor model (combining awareness and application), and a single-factor model. Initial analysis indicated that the eight-factor model demonstrated generally acceptable fit indices (χ^2^/df = 2.024, RMSEA = 0.041, CFI = 0.941, TLI = 0.933). However, the SRMR value of 0.133 exceeded the recommended threshold of 0.08, suggesting room for model refinement.

Based on modification index diagnostics and theoretical considerations, error covariance between two semantically similar items within the same dimension (AI evaluation) was freely estimated. Both items assessed capacity for critical scrutiny of AI outputs, rendering their error association theoretically justifiable. The revised model demonstrated substantially improved fit indices: χ^2^/df = 1.958, RMSEA = 0.040, CFI = 0.945, TLI = 0.937, SRMR = 0.072. All indices met or exceeded recommended thresholds (Hu and Bentler, 1999). The revised model significantly outperformed competing models in Table 6, indicating good discriminant validity among the core variables.

**Table 6:** Comparison of confirmatory factor analysis model fit indices (*N* = 600).

Model	χ^2^	df	χ^2^/df	RMSEA	CFI	TLI	SRMR
Eight-factor model	882.538	436	2.024	0.041	0.941	0.933	0.133
Seven-factor model^ᵃ^	1296.647	443	2.927	0.057	0.887	0.874	0.114
Single-factor model^ᵇ^	5183.209	464	11.171	0.130	0.378	0.335	0.268

ᵃ AI awareness and AI application were combined into a single factor.

ᵇ All items were loaded onto a single factor.

### 3.6 Structural equation modeling: direct effects testing

Structural equation modeling was employed to test research hypotheses H1a through H7c. The model demonstrated excellent fit: χ^2^/df = 1.277, CFI = 0.984, TLI = 0.982, RMSEA = 0.022, SRMR = 0.068, with all indices surpassing recommended thresholds. Detailed path coefficients are presented in Table 3 and illustrated in Figure 2.

In addition to path coefficients, the model’s explanatory power was assessed using squared multiple correlations (R^2^). The structural equation model explained 32.5% of the variance in learning motivation (R^2^ = 0.325). Specifically, the model explained 18.8% of the variance in autonomy (R^2^ = 0.188), 28.8% in competence (R^2^ = 0.288), and 39.9% in relatedness (R^2^ = 0.399). These R^2^ values indicate that the model has moderate to good explanatory power for learning motivation and the three basic psychological needs.

### 3.7 Mediation analysis

Bias-corrected bootstrap analysis with 5,000 resamples was employed to test the mediating effects of basic psychological needs. Results are presented in Table 7.

**Table 7:** Mediation effects of basic psychological needs (N = 600).

Hypothesis	Mediation path	Indirect effect (ab)	SE	Z	95% CI	Decision
H7a	AI Awareness → autonomy → learning motivation	0.048	0.016	3.06	[0.017, 0.079]	Supported
H7a	AI Application → autonomy → learning motivation	0.080	0.020	4.08	[0.042, 0.118]	Supported
H7b	AI evaluation → competence → learning motivation	0.143	0.031	4.61	[0.082, 0.204]	Supported
H7b	AI ethics → competence → learning motivation	0.135	0.028	4.75	[0.079, 0.191]	Supported
H7c	AI evaluation → relatedness → learning motivation	0.104	0.025	4.18	[0.055, 0.153]	Supported
H7c	AI ethics → relatedness → learning motivation	0.120	0.026	4.58	[0.069, 0.171]	Supported

SE, standard error; CI, confidence interval. All confidence intervals are bias-corrected and accelerated (BCa) bootstrap 95% CIs based on 5,000 resamples. CI excluding zero indicates significant mediation effect.

### 3.8 Qualitative findings

Thematic analysis of semi-structured interviews with 36 pre-service teachers and 8 course instructors revealed three major themes corresponding to the quantitative findings. Representative quotes are presented below to illustrate each theme. Participants were stratified into high-, middle-, and low-scoring groups based on the combined total score of AI literacy and learning motivation (see Section 2.2 for stratification details and Section 4.6 for discussion of this limitation).

#### 3.8.1 Cognitive outsourcing and autonomy diminution

Participants described a tendency to delegate cognitive tasks to AI, which they perceived as diminishing their independent thinking capabilities. A second-year pre-service teacher from the low-scoring group remarked:

"Now when I write lesson plans or prepare course materials, my first instinct is to have AI generate a framework, and then I just tweak it a bit. Over time, I feel like I'm increasingly incapable of independent thinking—it's as if I don't know where to start without AI."

Course instructors corroborated this observation from their professional perspective, noting that technical proficiency did not necessarily reflect pedagogical understanding:

"Some students extensively use AI-generated content in their internship lesson plans, but when you ask them 'why did you design it this way?', they often can't articulate the underlying pedagogical rationale. They're proficient at operating the tools, but their sense of autonomous control over the teaching process is clearly lacking."

#### 3.8.2 Critical mastery and competence affirmation

Students who engaged critically with AI outputs reported enhanced professional confidence and a strengthened sense of competence. A fourth-year pre-service teacher from the high-scoring group shared:

"There was a time when AI generated a lesson design that completely overlooked the practical constraints of rural schools. I drew on my internship experience to substantially revise it, and my supervising teacher praised the final product. In that moment, I felt an immense sense of accomplishment—I could genuinely sense my professional judgment improving."

The collaborative dimension of critical engagement also emerged, with students describing how collective deliberation around AI outputs strengthened peer connections:

One student from the high-scoring group noted: *"Our group frequently debates whether AI-generated content can be used directly, whether it needs modification, and how to revise it. These discussions have deepened my understanding of instructional design and strengthened my relationships with classmates."*

Instructors observed that students who engaged critically with AI demonstrated more coherent professional reasoning:

"Students who are willing to question AI outputs and engage in repeated deliberation and revision tend to present more coherently in group presentations and respond more effectively to peers' questions. Their professional confidence and collaborative rapport with classmates represent tangible growth."

#### 3.8.3 Professional identity and positive AI perceptions

Contrary to theoretical expectations that AI awareness might induce threat perceptions, pre-service teachers articulated a perspective in which AI proficiency was integrated into their emerging professional identity. A third-year pre-service teacher from the middle-scoring group explained:

"I pay attention to AI not because I think it's superior to me, but because I know I'll definitely need to use it as a future teacher. Understanding what it can and cannot do is about preparing in advance—it's not about comparing myself to it."

The collective learning context further shaped positive technological perceptions. A student from the middle-scoring group who participated in an AI education application workshop noted:

"Our group explored together how to integrate AI into elementary Chinese language instruction, learning and discussing as we went. Everyone's understanding of AI became clearer through these exchanges, rather than through solitary study. As a result, I actually felt I had more in common with my classmates."

Instructors confirmed that collaborative engagement with AI fostered social connections:

"Students who frequently participate in classroom discussions and willingly share their AI experiences tend to be more popular in class and more readily form learning circles. Their depth of AI understanding actually becomes a form of social capital."

#### 3.8.4 Negative/divergent cases and instructor perspectives

While the majority of students reported positive experiences with collaborative AI engagement, a small number of students (*n* = 3) expressed concerns that diverged from the main themes. One student from the middle-scoring group noted: “Sometimes I feel that group discussions about AI can be superficial because everyone just agrees with each other. I learn more when I struggle with AI alone and then ask for help.” This divergent case suggests that the benefits of collaborative engagement may depend on the quality of group interaction, not merely its presence.

Instructors offered complementary and sometimes more critical perspectives. One instructor observed: “I notice that some students use AI as a crutch rather than a tool. They become dependent very quickly.” Another instructor noted: “The students who benefit most from AI are those who already have strong foundational knowledge. For weaker students, AI can actually widen the gap.” These instructor perspectives provide an external lens that both corroborates and extends the student-reported experiences.

## 4 Discussion

This study examined the associations among the four-dimensional structure of AI literacy, basic psychological needs, and pre-service teachers’ learning motivation. Drawing on self-determination theory (Deci and Ryan, 1985) and employing an explanatory sequential mixed-methods design, we tested a theoretical model integrating the differentiated associations of AI awareness, application, evaluation, and ethics. The findings revealed three major insights: (1) AI literacy dimensions showed sharply differentiated associations with basic psychological needs, with awareness and application showing negative associations with autonomy while evaluation and ethics showed positive associations with competence and relatedness; (2) all three psychological needs showed significant mediating roles in the associations between AI literacy and learning motivation, with competence demonstrating the strongest indirect effects; and (3) contrary to technological threat theories, AI awareness showed positive associations with both competence and relatedness among pre-service teachers. The authors note that causal directionality cannot be determined from the cross-sectional design.

### 4.1 Differential effects of AI literacy on basic psychological needs

#### 4.1.1 Negative effects of awareness and application on autonomy

Consistent with H1a and H2, both AI awareness (*β* = −0.190, *p* < 0.001) and AI application (*β* = −0.332, *p* < 0.001) significantly and negatively predicted pre-service teachers’ autonomy. These findings confirm the applicability of the Cognitive Miser Principle (Fiske and Taylor, 1991) and automation bias theory (Parasuraman and Manzey, 2010) within intelligent learning contexts. When learners possess heightened awareness of AI’s capabilities and advanced skills in applying AI tools, they may be predisposed to minimize cognitive effort and place excessive trust in automated systems, thereby undermining their sense of volitional control over the learning process.

This pattern aligns closely with Watts (2025) concept of “cognitive debt,” which suggests that excessive reliance on AI to complete higher-order cognitive tasks may be associated with gradual atrophy of autonomous thinking capabilities through lack of practice. Romeo and Conti's (2025) systematic review similarly identified passive engagement as a key risk factor for automation bias, noting that active user engagement appears essential for maintaining critical thinking. The qualitative findings in Section 3.8.1 illuminate this mechanism: students described their “first instinct” as turning to AI for lesson planning, and instructors observed that students proficient in AI tools often struggled to articulate the pedagogical rationale behind their designs. These accounts suggest that technical proficiency does not automatically translate into learning autonomy; rather, when AI transforms from “tool” to “surrogate,” the sense of autonomy diminishes.

The stronger negative association for AI application (*β* = −0.332) than for AI awareness (β = −0.190) warrants attention. This differential magnitude suggests that active deployment of AI Application may pose a greater risk to autonomy than mere awareness. The qualitative finding of “cognitive outsourcing” (Section 3.8.1) illuminates this mechanism. Students described their “first instinct” to delegate lesson planning to AI, with one stating, “I feel like I’m increasingly incapable of independent thinking—it’s as if I don’t know where to start without AI.” This suggests that repeated application may produce a habit of cognitive delegation that gradually atrophies autonomous thinking, whereas passive awareness does not necessarily trigger this pattern. Instructors’ observations that technically proficient students often “can’t articulate the underlying pedagogical rationale” further support this interpretation: the negative association reflects displacement of autonomous reasoning by automated generation, not lack of technical skill. This interpretation is consistent with Watts (2025) concept of cognitive debt, which accrues through repeated delegation of cognitive tasks rather than through passive knowledge of technological capabilities.

#### 4.1.2 Positive effects of evaluation and ethics on competence and relatedness

As hypothesized in H3 through H6, AI evaluation and AI ethics demonstrated significant positive associations with both competence and relatedness. AI evaluation positively predicted competence (*β* = 0.315, *p* < 0.001) and relatedness (*β* = 0.333, *p* < 0.001), while AI ethics positively predicted competence (*β* = 0.326, *p* < 0.001) and relatedness (*β* = 0.419, *p* < 0.001). These findings are consistent with and extend prior research on the empowering potential of critical AI engagement.

The positive association between AI evaluation and competence aligns with Bećirović et al.'s (2025) finding that students capable of effectively evaluating AI outputs report significantly higher self-efficacy. This pattern suggests that when learners identify and correct deviations in AI outputs, their professional judgment may receive immediate validation through authentic tasks—a critical source of competence satisfaction within self-determination theory (Deci and Ryan, 1985; Son et al., 2025). The qualitative evidence in Section 3.8.2 illustrates this mechanism: a fourth-year student described how revising AI-generated lesson content based on internship experience led to supervisor praise and an “immense sense of accomplishment,” reflecting enhanced professional competence.

The positive association between AI evaluation and relatedness is consistent with and extends Funa and Gabay's (2025) research, which identified cognitive criticality as the strongest predictor of teaching integration intentions among pre-service teachers. The present findings suggest that critical evaluation may not only shape pedagogical motivation but also be associated with stronger social connections. As revealed in Section 3.8.2, students described how group debates about AI output quality “deepened my understanding of instructional design and strengthened my relationships with classmates.” This aligns with Chiu et al.'s (2023) conceptualization of collaborative evaluation as knowledge co-construction practice that enhances cohesion within learning communities.

AI ethics demonstrated the strongest positive effect among all predictors, particularly on relatedness (β = 0.419). This finding supports social identity theory (Tajfel and Turner, 1986): learners who adhere to AI ethical norms are more likely to receive positive social recognition from instructors and peers, thereby strengthening professional identity and group belonging. Yi and Siquan (2025) similarly found that satisfaction of basic psychological needs, mediated by AI literacy and self-efficacy, significantly influences pre-service teachers’ behavioral intentions to use AI. The present results extend this work by identifying ethical engagement as a particularly potent source of relatedness satisfaction. Solyst et al.'s (2025) observation that critical examination of AI ethical limitations reinforces professional identity through communal deliberation resonates with this pattern.

### 4.2 Mediating role of basic psychological needs

Consistent with H7a through H7c, all three basic psychological needs showed significant mediating roles in the associations between AI literacy dimensions and learning motivation. Autonomy was found to mediate the associations between AI awareness and learning motivation (indirect effect = 0.048, 95% CI [0.017, 0.079]) and between AI application and learning motivation (indirect effect = 0.080, 95% CI [0.042, 0.118]). Competence mediated the associations between AI evaluation and learning motivation (indirect effect = 0.143, 95% CI [0.082, 0.204]) and between AI ethics and learning motivation (indirect effect = 0.135, 95% CI [0.079, 0.191]). Relatedness mediated the associations between AI evaluation and learning motivation (indirect effect = 0.104, 95% CI [0.055, 0.153]) and between AI ethics and learning motivation (indirect effect = 0.120, 95% CI [0.069, 0.171]).

These findings are consistent with the core proposition of self-determination theory within intelligent learning contexts: environmental factors (here, AI literacy dimensions) may influence intrinsic motivation indirectly through their associations with satisfaction or frustration of basic psychological needs (Deci and Ryan, 1985; Ryan and Deci, 2020). The pattern of mediation aligns closely with Huang and Zhao's (2025) finding that AI literacy is associated with job satisfaction among university faculty through psychological need satisfaction, and Tulis et al.'s (2025) demonstration that challenge appraisal predicts AI usage intentions via autonomy and competence.

Notably, competence exhibited the strongest indirect effects (0.135–0.143) among the three mediators. Competence satisfaction surpasses autonomy and relatedness in importance within AI learning contexts, emerging as the central node connecting the AI motivational system. The predominance of competence as a mediator suggests that for pre-service teachers, the affirmation of professional capability constitutes the primary psychological pathway through which technological literacy translates into motivational outcomes. This interpretation is consistent with the qualitative accounts in Section 3.8.2, where students described how critical engagement with AI outputs validated their professional judgment and enhanced their sense of accomplishment.

### 4.3 Positive associations between AI awareness and competence and relatedness: the identity-context dual boundary effect

One of the most notable findings of this study was that AI awareness positively predicted both competence (*β* = 0.142, *p* = 0.005) and relatedness (*β* = 0.172, *p* < 0.001). This finding was unexpected, as H1b and H1c were exploratory and did not specify a direction. This pattern contradicts the negative predictions derived from social comparison theory (Festinger, 1954) and technostress theory (Tarafdar et al., 2007), which suggest that awareness of powerful technological capabilities may induce upward comparison, ability anxiety, and psychological burden (Brougham and Haar, 2018; Turkle, 2015). However, cross-sectional data cannot determine whether these positive associations reflect causal influences or other unmeasured factors.

Notably, AI awareness showed divergent effects: it negatively predicted autonomy but positively predicted competence and relatedness. This duality is not contradictory. The negative effect on autonomy reflects the Cognitive Miser Principle—awareness of AI’s capabilities triggers cognitive offloading, reducing volitional control. The positive effects on competence and relatedness, however, depend on identity and contextual moderators: when pre-service teachers view AI as integral to their professional future and learn collaboratively, awareness becomes empowering rather than threatening.

The qualitative evidence in Section 3.8.3 illuminates the mechanisms underlying this unexpected finding. First, pre-service teachers’ dual identity as both learners and future educators facilitated a value shift in their technological awareness. Unlike general university students, pre-service teachers conceptualize AI proficiency as integral to their emerging professional competence rather than as a threat to their personal capabilities. As one participant articulated, “I pay attention to AI not because I think it’s superior to me, but because I know I’ll definitely need to use it as a future teacher.” This career-oriented cognitive perspective transforms heightened technological awareness into a manifestation of professional development motivation.

Second, technological awareness developed within collective learning contexts. The qualitative accounts revealed that AI literacy education in teacher training institutions typically employs collaborative pedagogical models, wherein technological awareness is formed and deepened through group inquiry and shared exploration. Students described how learning and discussing AI applications together made their understanding clearer and actually strengthened their connections with classmates. Instructors observed that students who actively shared AI experiences tended to form learning circles, with their depth of AI understanding becoming “a form of social capital.”

Synthesizing these patterns, we propose an “identity-context dual boundary” theoretical framework to account for the unexpected positive effects. The directional influence of AI awareness on basic psychological needs is jointly moderated by two boundary conditions: (1) the identity boundary—whether learners frame AI as constitutive of professional competence versus threatening to personal ability; and (2) the contextual boundary—whether technological awareness develops within collaborative versus isolated use contexts. When learners cross these boundaries—framing AI as integral to professional identity and developing awareness through collaborative engagement—AI awareness transforms from a potential source of psychological burden into a source of psychological empowerment.

**Figure 3:**
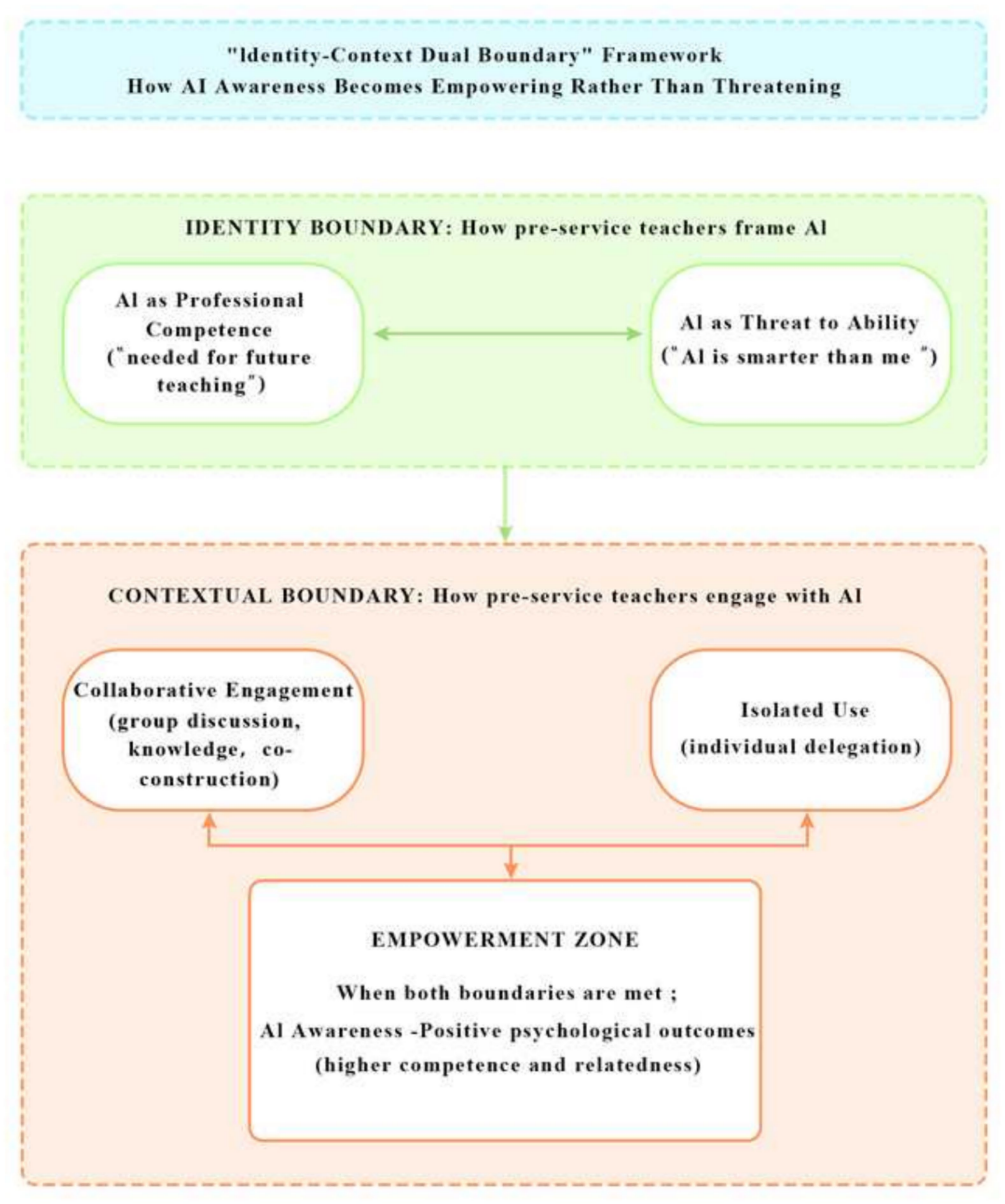
The identity-context dual boundary framework. The framework proposes that AI awareness becomes empowering when pre-service teachers frame AI as integral to professional identity (identity boundary) and develop awareness through collaborative engagement (contextual boundary).

This theoretical proposition, if supported by future longitudinal research, would refine the applicability boundaries of Cognitive Miser Principle and social alienation theory within intelligent educational contexts for pre-service teacher populations. It resonates with Tulis et al.'s (2025) concept of “challenge appraisal” and is consistent with Son et al.'s (2025) finding that AI feedback is associated with learning engagement through autonomy support and competence confirmation, while identifying the specific conditions under which AI awareness may be associated with empowerment rather than threat. Figure 3 illustrates this proposed framework, showing how the interaction between identity and contextual boundaries determines whether AI awareness becomes empowering or threatening.

This “identity-context dual boundary” framework, therefore, refines the boundary conditions of social comparison theory and technostress theory. It suggests that these theories’ predictions of negative psychological outcomes (e.g., anxiety, alienation) are not universal, but rather hold primarily when individuals lack a strong professional identity that integrates the technology and when they engage with it in isolation. By specifying the conditions under which AI awareness becomes empowering, our framework provides a more nuanced and contextualized understanding of human-AI interaction in educational settings.

### 4.4 Theoretical contributions

This study offers several theoretical contributions to the literature on AI literacy, self-determination theory, and learning motivation, while acknowledging that causal claims await validation through longitudinal designs.

First, by disaggregating AI literacy into its four constituent dimensions—awareness, application, evaluation, and ethics—this study demonstrates that these dimensions exert fundamentally different, and even opposing, effects on basic psychological needs. This finding challenges the prevalent tendency in AI literacy research to conceptualize the construct as unidimensional (e.g., Bewersdorff et al., 2025) and underscores the importance of dimensional specificity in understanding how technological literacy shapes psychological outcomes.

Second, this study extends the explanatory scope of self-determination theory to intelligent educational contexts. While prior research has established the mediating role of basic psychological needs in various learning environments (Van den Broeck et al., 2016), the present findings demonstrate that SDT’s core mechanisms operate similarly when the environmental factor is technological literacy. Moreover, the identification of competence as the strongest mediator suggests that competence affirmation may be particularly salient in AI-mediated learning.

Third, this study refines the boundary conditions of Cognitive Miser Principle (Fiske and Taylor, 1991), automation bias theory (Parasuraman and Manzey, 2010), and social alienation theory (Turkle, 2015) for pre-service teacher populations. The proposed “identity-context dual boundary” framework specifies the conditions under which AI awareness transitions from psychological burden to psychological resource, thereby reconciling the apparent contradiction between technological threat theories and the present findings. This framework contributes to a more nuanced understanding of how population characteristics and learning contexts moderate the psychological effects of technological awareness.

Fourth, the integration of quantitative path analysis with qualitative thematic analysis provides methodological triangulation that enhances confidence in the findings. The qualitative accounts not only illustrated the statistical patterns but also revealed the underlying mechanisms—professional identity formation and collaborative knowledge co-construction—that explain the unexpected positive effects of AI awareness. This mixed-methods approach addresses calls for richer understanding of the psychological processes underlying technology-mediated learning (Chiu et al., 2023).

### 4.5 Practical implications

The findings of this study have several practical implications for teacher education institutions seeking to cultivate AI literacy while supporting pre-service teachers’ learning motivation.

*Four-Dimensional Synergy in Curriculum Design*. AI literacy curricula should be designed with an overarching framework that positions evaluation and ethics as the core, with awareness and application as the foundation. Lower-division courses should emphasize awareness cultivation and application training, integrating autonomy development into foundational skill instruction through tasks such as human-AI collaborative comparison and AI output revision. These activities can help students objectively recognize technology’s instrumental nature while maintaining active cognitive engagement. Upper-division courses should shift focus toward evaluation and ethics, offering advanced seminars in AI educational evaluation, educational ethics, and discipline-specific integration to enhance students’ critical mastery of technology. The curriculum may further incorporate disciplinary modules that leverage domain-specific evaluation criteria: mathematics and natural sciences emphasizing data analysis and model logic, humanities and social sciences focusing on textual evaluation and value judgment, and arts and physical education attending to creative design and ethical boundaries.

*Needs-Oriented Pedagogical Approaches*. Instructional design should prioritize satisfaction of pre-service teachers’ basic psychological needs. Addressing the tendency for autonomy diminution during awareness and application learning, problem-oriented inquiry-based teaching may be employed—organizing instruction around authentic problems such as “the boundaries of AI application in rural education”—enabling students to maintain autonomous decision-making throughout problem-solving processes. For cultivating competence and relatedness, group collaboration and learning community models prove more effective: through tasks such as AI educational application design and collective evaluation of AI outputs, students receive competence affirmation through collaboration while establishing deep connections through professional discourse. The qualitative findings in Section 3.8.2 and 3.8.3 underscore the importance of collaborative learning contexts in transforming AI awareness from potential threat into social capital.

*Contextual Embedding Across Authentic Practice*. Literacy cultivation cannot be confined to classroom instruction but must extend into authentic contexts including educational internships, micro-teaching, and professional practicums. Students should engage in repeated application of evaluation and ethics competencies throughout the full cycle of instructional design, implementation, and reflection. As Funa and Gabay (2025) noted, such contextualized literacy application constitutes the critical juncture where technological awareness transforms into pedagogical capability. Concurrently, behavioral guidelines for pre-service teachers’ AI use should be established, delineating appropriate boundaries across different task contexts: encouraging AI utilization for lower-order cognitive tasks such as information organization and idea generation, while discouraging direct application to higher-order cognitive tasks such as core thesis writing or original complete instructional design. Such guidelines can guide students toward moderate and autonomous usage habits cultivated through reasoned reflection.

### 4.6 Limitations and future directions

This study has several limitations that should be acknowledged, which also delineate avenues for future investigation.

First, the quantitative component employed a cross-sectional survey design. While this approach was appropriate for establishing the factor structure and correlational patterns of AI literacy among pre-service teachers, cross-sectional data cannot determine causal directionality—whether AI literacy dimensions influence learning motivation, whether motivated students are more likely to develop AI literacy, or whether reciprocal relationships exist. A longitudinal design was not feasible within the one-year project timeline, and the absence of prior longitudinal data on this population meant that appropriate measurement intervals could not be determined *a priori*. Future research should employ cross-lagged panel models tracking pre-service teachers over three time points (e.g., beginning, middle, and end of an academic year) to test reciprocal relationships, such as whether AI evaluation ability predicts subsequent competence satisfaction and vice versa. Additionally, drawing upon Son et al.'s (2025) longitudinal intervention design, future studies could use randomized controlled trials with latent growth curve modeling to examine trajectories of change in psychological need satisfaction and learning motivation following AI literacy training.

Second, the sampling scope was limited to a single institution—Northeast Normal University—excluding pre-service teachers from central and western regional teacher education institutions, vocational teacher training programs, and master’s-level education students. The generalizability of findings requires further validation across more diverse populations, thereby testing the cross-group applicability of the “identity-context dual boundary” effect. Future studies should recruit participants from multiple institutions across different regions and educational contexts.

Third, the self-report nature of the quantitative measures introduces the possibility of social desirability bias, particularly for constructs such as AI ethics. While Harman’s single-factor test suggested that common method bias was not a serious concern, future research could incorporate behavioral measures or objective indicators of AI literacy (e.g., performance-based tasks) to complement self-report data.

Fourth, this study did not conduct intervention experiments. Although the proposed cultivation pathways are theoretically grounded, their effectiveness requires empirical validation. Future research should design intervention programs based on the four-dimensional synergistic framework identified in this study, employing randomized controlled trials or quasi-experimental designs to validate their effectiveness in enhancing AI literacy, psychological need satisfaction, and learning motivation. Such interventions would complete the cycle from theory to practice, providing actionable evidence for teacher education curriculum reform.

Fifth, the qualitative component, while rich in experiential data, was limited to a single time point. Longitudinal qualitative designs following pre-service teachers through their teacher preparation programs could reveal how perceptions of AI and associated psychological need satisfaction evolve over time, particularly as students transition from coursework to student teaching and ultimately to professional practice.

Sixth, the qualitative sampling strategy combined AI literacy and learning motivation scores into a single total score for stratification, rather than using a 2 × 2 factorial design (high/low AI literacy × high/low learning motivation). This conflates the independent variable (AI literacy) and dependent variable (learning motivation) and may systematically exclude discordant cases (e.g., high AI literacy with low motivation). Future qualitative research should employ a 2 × 2 stratified sampling matrix based on theoretically separate dimensions.

## 5 Conclusion

Grounded in self-determination theory and employing an explanatory sequential mixed-methods design incorporating survey data from 600 pre-service teachers and semi-structured interviews with 36 pre-service teachers and 8 course instructors, this study systematically examined the mechanisms through which the four-dimensional structure of AI literacy influences pre-service teachers’ learning motivation. The findings, while consistent with a mediation model, yield three core conclusions, though causal directionality cannot be determined from the cross-sectional data.

First, the four dimensions of AI literacy showed sharply differentiated associations with basic psychological needs. Specifically, AI awareness and application were negatively associated with autonomy (*β* = −0.190 and −0.332, respectively), consistent with the Cognitive Miser Principle and automation bias theory. In contrast, AI evaluation and ethics were positively associated with competence (β = 0.315 and 0.326) and relatedness (β = 0.333 and 0.419), supporting the critical mastery perspective.

Second, all three basic psychological needs played significant mediating roles in the relationship between AI literacy and learning motivation, with competence exhibiting the strongest indirect effects (0.135–0.143). This finding collectively points to a core insight: competence confirmation constitutes the critical psychological pathway through which technological literacy translates into motivational outcomes. The qualitative accounts in Section 3.8.2 illustrated this mechanism, as students described how critical engagement with AI outputs validated their professional judgment and enhanced their sense of accomplishment.

Third, an unexpected finding emerged: AI awareness demonstrated weak but significant positive associations with both competence (*β* = 0.142, *p* = 0.005) and relatedness (*β* = 0.172, *p* < 0.001). Qualitative analysis indicated that this result is closely related to pre-service teachers’ dual identity—they perceive AI proficiency as constitutive of future teachers’ professional competence rather than as a threat to their current personal capabilities. Concurrently, collaborative group learning contexts involving knowledge co-construction reinforced this cognitive transformation. Based on these findings, this study proposes an “identity-context dual boundary” theoretical framework: when learners frame AI as constitutive of professional competence (identity boundary) and technological perceptions develop within collaborative contexts (contextual boundary), AI awareness may transform from a source of psychological burden into a source of psychological empowerment.

Theoretically, this investigation extends the explanatory scope of self-determination theory within intelligent educational contexts and refines the boundary conditions of Cognitive Miser Principle and social alienation theory for pre-service teacher populations. By disaggregating AI literacy into its four constituent dimensions, the study demonstrates that these dimensions exert fundamentally different psychological effects, challenging the prevalent tendency to conceptualize AI literacy as a unidimensional construct. The proposed identity-context dual boundary framework specifies the conditions under which AI awareness transitions from psychological burden to psychological resource, reconciling the apparent contradiction between technological threat theories and the present findings.

Practically, these results provide empirical foundations for developing AI literacy curricula in teacher education that are psychologically needs-oriented and synergistically integrated across four competency dimensions. Teacher education institutions should design curricula that position evaluation and ethics as the core, with awareness and application as the foundation; adopt pedagogical approaches that prioritize satisfaction of autonomy, competence, and relatedness; and embed literacy development across authentic practice contexts including educational internships and professional practicums. Such psychologically informed curriculum design can help cultivate pre-service teachers who not possess technical proficiency but also maintain autonomous agency and intrinsic motivation in an increasingly AI-mediated educational landscape.

## Data Availability

The original contributions presented in the study are included in the article/Supplementary material, further inquiries can be directed to the corresponding author.

## References

[ref1] Ayanwale M. A. Adelana O. P. Molefi R. R. Adeeko O. Ishola A. M. (2024). Examining artificial intelligence literacy among pre-service teachers for future classrooms. Comput. Educ. Open 6:100179. doi: 10.1016/j.caeo.2024.100179

[ref2] Bećirović S. Polz E. Tinkel I. (2025). Exploring students' AI literacy and its effects on their AI output quality, self-efficacy, and academic performance. Smart Learn. Environ. 12:29. doi: 10.1186/s40561-025-00384-3

[ref3] Bewersdorff A. Hornberger M. Nerdel C. Schiff D. S. (2025). AI advocates and cautious critics: how AI evaluation, AI interest, use of AI, and AI literacy build university students' AI self-efficacy. Comput. Educ. 8:100340. doi: 10.1016/j.caeai.2024.100340

[ref9001] Braun V. Clarke V. (2006). Using thematic analysis in psychology. Qual. Res. Psychol. 3, 77–101. doi: 10.1191/1478088706qp063oa

[ref4] Brougham D. Haar J. (2018). Smart technology, artificial intelligence, robotics, and algorithms (STARA): employees' perceptions of our future workplace. J. Manag. Organ. 24, 239–257. doi: 10.1017/jmo.2016.55

[ref5] Bruner J. S. (1960). The Process of Education. Cambridge, MA: Harvard University Press.

[ref6] Chiu T. K. F. Xia Q. Zhou X. Chai C. S. Cheng M. (2023). Systematic literature review on opportunities, challenges, and future research recommendations of artificial intelligence in education. Comput. Educ. 4:100118. doi: 10.1016/j.caeai.2022.100118

[ref7] Chomsky N. (1965). Aspects of the Theory of Syntax. Cambridge, MA: MIT Press.

[ref8] Deci E. L. Ryan R. M. (1985). Intrinsic Motivation and Self-Determination in Human Behavior. New York: Plenum Press.

[ref9] Festinger L. (1954). A theory of social comparison processes. Hum. Relat. 7, 117–140. doi: 10.1177/001872675400700202

[ref10] Fiske S. T. Taylor S. E. (1991). Social Cognition. 2nd Edn New York: McGraw-Hill.

[ref11] Funa A. A. Gabay R. A. (2025). Are pre-service teachers ready to teach the alpha generation? The impact of pre-service teachers' ChatGPT literacy levels on behavioral intentions. Comput. Educat. 8:100362. doi: 10.1016/j.caeai.2025.100362

[ref12] Gottfried A. E. (1985). Academic intrinsic motivation in elementary and junior high school students. J. Educ. Psychol. 77, 631–645. doi: 10.1037/0022-0663.77.6.631

[ref9002] Hu L. T. Bentler P. M. (1999). Cutoff criteria for fit indexes in covariance structure analysis: conventional criteria versus new alternatives. Struct. Equ. Model. 6, 1–5. doi: 10.1080/10705519909540118, 41040088

[ref13] Huang L. Zhao Y. (2025). The impact of AI literacy on work-life balance and job satisfaction among university faculty: a self-determination theory perspective. Front. Psychol. 16:1669247. doi: 10.3389/fpsyg.2025.1669247, 41040088 PMC12487956

[ref14] Long D. Magerko B. (2020). What is AI literacy? Competencies and design considerations. In Proceedings of the 2020 CHI Conference on Human Factors in Computing Systems (pp. 1–16). New York: ACM.

[ref15] Ministry of Education of the People's Republic of China (2022). Teacher Digital Literacy (JY/T 0646–2022). Beijing: Ministry of Education of the People's Republic of China.

[ref16] Ministry of Education of the People's Republic of China. (2024) The 2024 National Education Work Conference held in Beijing. Available online at: http://www.moe.gov.cn/jyb_xwfb/gzdt_gzdt/moe_1485/202401/t20240111_1099814.html (Accessed March 14, 2026).

[ref17] Ng D. T. K. Leung J. K. L. Chu S. K. W. Qiao M. S. (2021). Conceptualizing AI literacy: an exploratory review. Comput. Educ. Art. Intellig. 2:100041. doi: 10.1016/j.caeai.2021.100041

[ref18] OECD (2003). Key Competencies for a Successful Life and a Well-Functioning Society. Göttingen: Hogrefe & Huber.

[ref19] Parasuraman R. Manzey D. H. (2010). Complacency and bias in human use of automation: an attentional integration. Hum. Factors 52, 381–410. doi: 10.1177/0018720810376055 21077562

[ref20] Podsakoff P. M. MacKenzie S. B. Lee J. Y. Podsakoff N. P. (2003). Common method biases in behavioral research: a critical review of the literature and recommended remedies. J. Appl. Psychol. 88, 879–903. doi: 10.1037/0021-9010.88.5.879, 14516251

[ref21] Romeo G. Conti D. (2025). Exploring automation bias in human–AI collaboration: a review and implications for explainable AI. AI Soc. 41, 259–278. doi: 10.1007/s00146-025-02422-7

[ref22] Ryan R. M. Deci E. L. (2020). Intrinsic and extrinsic motivation from a self-determination theory perspective: definitions, theory, practices, and future directions. Contemp. Educ. Psychol. 61:101860. doi: 10.1016/j.cedpsych.2020.101860

[ref23] Solyst J. Pan M. Y. Andam A. Poblete I. P. Eslami M. Hammer J. . (2025). Critical AI literacy through exploring generative AI limitations. In Rajala A. Cortez A. Hofmann R. Jornet A. Lotz-Sisitka H. Markauskaite L. (Eds.), Proceedings of the 19th International Conference of the Learning Sciences (ICLS 2025) (pp. 2061–2065). International Society of the Learning Sciences.

[ref24] Son M. Ružić I. Philpott C. (2025). The role of AI-driven feedback in fostering growth mindset and engagement: a self-determination theory perspective. Learn. Motiv. 92:102192. doi: 10.1016/j.lmot.2025.102192

[ref25] Tajfel H. Turner J. C. (1986). “The social identity theory of intergroup behavior,” in Psychology of Intergroup Relations, eds. Worchel S. Austin W. G.. 2nd ed (Chicago: Nelson-Hall), 7–24.

[ref26] Tarafdar M. Tu Q. Ragu-Nathan B. S. Ragu-Nathan T. S. (2007). The impact of technostress on role stress and productivity. J. Manag. Inf. Syst. 24, 301–328. doi: 10.2753/MIS0742-1222240109

[ref27] Tulis M. Brandhofer G. Tengler K. (2025). Motivational and appraisal factors shaping generative AI use and intention in Austrian higher education students and teachers. Front. Educ. 10:1677827. doi: 10.3389/feduc.2025.1677827

[ref28] Turkle S. (2015). Reclaiming Conversation: The Power of Talk in a Digital Age. New York: Penguin Press.

[ref29] UNESCO (2024). AI Competency Framework for Students. Paris: UNESCO.

[ref30] Van den Broeck A. Ferris D. L. Chang C. H. Rosen C. C. (2016). A review of self-determination theory's basic psychological needs at work. J. Manag. 42, 1195–1229. doi: 10.1177/0149206316632058

[ref31] Van den Broeck A. Vansteenkiste M. De Witte H. Soenens B. Lens W. (2010). Capturing autonomy, competence, and relatedness at work: construction and initial validation of the work-related basic need satisfaction scale. J. Occup. Organ. Psychol. 83, 981–1002. doi: 10.1348/096317909X481382

[ref32] Watts K. J. (2025). Paying the cognitive debt: an experiential learning framework for integrating AI in social work education. Educ. Sci. 15:1304. doi: 10.3390/educsci15101304

[ref33] White R. W. (1959). Motivation reconsidered: the concept of competence. Psychol. Rev. 66, 297–333. doi: 10.1037/h0040934, 13844397

[ref34] Wut T. M. Wong H. Y. Lee D. (2025). School support, basic psychological needs, and AI literacy development among teachers. Educ. Inform. Technol. 30, 16291–16320. doi: 10.1007/s10639-025-13456-1

[ref35] Yi Z. Siquan X. (2025). The impact of pre-service language teachers' basic psychological needs on behavioural intentions to utilise artificial intelligence in teaching: AI literacy and self-efficacy as mediators. Eur. J. Educ. 60:e70160. doi: 10.1111/ejed.70160

[ref36] Yuan P. L. Chen Y. Z. Wang X. Z. Song H. (2025). "Expectation" or "threat": an empirical analysis of pre-service teachers' AI awareness types and their TPACK level differences. Teach. Educ. Res. 37, 24–32.

