# Supplementary material for: The influence of AI literacy on pre-service teachers’ learning motivation: the mediating role of basic psychological needs
Source: Front Psychol. 2026 May 8;17:1831770. doi: 10.3389/fpsyg.2026.1831770 (PMC13194449; doi:10.3389/fpsyg.2026.1831770)
Supplement: Supplementary file 3 [file Table_1.DOCX]

**Appendix A**

**A1. Pilot Study Questionnaire (N = 304)**

The pilot study was conducted from April to May 2025 with an independent sample of 304 pre-service teachers who did not participate in the formal survey or qualitative interviews. All items were measured on a 5-point Likert scale (1 = strongly disagree, 5 = strongly agree). Reverse-scored items (R) were transformed prior to analysis.

**Part I: Demographic Information**

| ****Variable**** | ****Category**** | ****Options**** |
| --- | --- | --- |
| Gender | 性别 | □ Male 男 □ Female 女 |
| Grade Level | 年级 | □ First year 大一 □ Second year 大二 □ Third year 大三 □ Fourth year 大四 |
| Academic Discipline | 专业类型 | □ Humanities and Social Sciences 人文社科类师范 □ Mathematics and Natural Sciences 数理科学类师范 □ Arts and Physical Education 艺术体育类师范 □ Other (please specify) 其他师范（请注明） |
| AI Usage Frequency | AI工具使用频率 | □ Almost never 几乎不用 □ 1-3 times per month 每月1-3次 □ 1-3 times per week 每周1-3次 □ Almost every day 几乎每天使用 |

****Part II: AI Literacy and Psychological Needs Scale****

| ****Item No.**** | ****Dimension**** | ****English**** | ****中文**** |
| --- | --- | --- | --- |
| 1 | Awareness | I know which AI tools can be used for my professional studies and teaching preparation. | 我了解哪些AI工具可以用于我的专业学习和教学准备 |
| 2 | Awareness | I actively follow new developments and application cases of AI technology in education. | 我会主动关注AI技术在教育领域的新发展和应用案例 |
| 3 | Awareness | I can clearly recognize the impacts and challenges that AI technology brings to the future teaching profession. | 我能清晰认识到AI技术对未来教师职业带来的影响和挑战 |
| 4 | Awareness (R) | After learning about AI's capabilities, I sometimes reduce active thinking about improving my own professional competence. | 了解AI的强大能力后，我有时会减少对提升自身专业能力的主动思考（R） |
| 5 | Application | I can skillfully use AI tools to generate lesson plans, course materials, or learning activity suggestions. | 我能熟练使用AI工具来生成教案、课件或学习活动建议 |
| 6 | Application | I can use AI tools to analyze and process teaching-related data or textual materials in my discipline. | 我能利用AI工具分析和处理与专业相关的教学数据或文本材料 |
| 7 | Application | I can flexibly adjust how I use AI tools and their outputs based on actual teaching needs. | 我能根据实际教学需求，灵活调整AI工具的使用方式和输出内容 |
| 8 | Application (R) | When facing complex teaching tasks, I tend to rely on AI to generate complete solutions rather than conceiving on my own. | 遇到复杂的教学任务，我更倾向于依赖AI生成完整方案，而不是自己构思（R） |
| 9 | Evaluation | I evaluate whether AI-generated content is accurate, reasonable, and appropriate. | 我会判断AI生成的内容是否准确、合理、适用 |
| 10 | Evaluation | Before adopting AI suggestions, I make judgments based on my professional knowledge. | 在采纳AI的建议前，我会结合自己的专业知识进行判断 |
| 11 | Evaluation | I adapt AI-generated content based on actual teaching situations before using it. | 我会根据实际教学情况，对AI生成的内容进行调整后使用 |
| 12 | Evaluation (R) | I find it difficult to identify errors in AI outputs. | 我很难能识别AI输出中的错误（R） |
| 13 | Ethics | When using AI to complete assignments or design lesson plans, I pay attention to avoiding academic misconduct such as plagiarism. | 用AI完成作业或设计教案时，我会注意避免抄袭等学术不端行为 |
| 14 | Ethics | I am aware of and take seriously the copyright, privacy, and data security issues that may arise when using AI. | 我能意识到并重视使用AI时可能涉及的版权、隐私和数据安全问题 |
| 15 | Ethics | Adhering to AI usage norms makes me feel like a responsible professional learner. | 遵守AI的使用规范，让我觉得自己是一个负责任的专业学习者 |
| 16 | Ethics (R) | I believe that occasionally copying AI-generated content directly for assignments does not affect academic integrity. | 我认为偶尔直接复制AI生成的内容来完成作业，不会影响学术诚信（R） |
| 17 | Autonomy | Knowing what AI can do makes me feel that I have more paths and resources to explore in my professional learning. | 了解AI能做什么之后，我觉得自己在专业学习中可探索的路径和资源更丰富了 |
| 18 | Autonomy | When using AI to assist learning, I can interact with it at my own pace and in my own way. | 使用AI辅助学习时，我可以按照自己的节奏和方式来与它互动 |
| 19 | Autonomy (R) | When learning with AI, I sometimes unconsciously follow AI's way of thinking instead of actively thinking on my own. | 使用AI学习时，我有时会不自觉地跟随AI的思路，而不是自己主动思考（R） |
| 20 | Autonomy | Even if AI's suggestions sound reasonable, I still insist on my own judgment if they conflict with my ideas. | 即使AI的建议听起来合理，如果与我的想法不一致，我仍会坚持自己的判断 |
| 21 | Competence | Knowing that AI can assist me gives me more confidence to complete challenging professional learning goals. | 知道AI可以辅助我之后，我更有信心完成有挑战性的专业学习目标 |
| 22 | Competence | By using AI to solve professional problems, I feel that my ability to handle complex teaching issues has improved. | 通过使用AI解决专业难题，我感觉自己处理复杂教学问题的能力有所提升 |
| 23 | Competence | Being able to identify and correct AI's mistakes makes me feel that I have professional judgment. | 我能识别并修正AI的错误，这让我觉得自己具备专业判断力 |
| 24 | Competence (R) | After using AI, I feel that my professional competence has not substantially improved. | 使用AI之后，我觉得自己的专业能力并没有得到实质性提升（R） |
| 25 | Relatedness | Discussing with classmates how AI will change future teaching makes me feel that we are facing an important challenge together, bringing us closer. | 和同学一起讨论AI如何改变未来教学，让我感受到我们正在共同面对一项重要挑战，彼此关系更紧密了 |
| 26 | Relatedness | The experience of using AI together to solve problems in group tasks has enhanced our team cohesion. | 在小组任务中共同使用AI解决问题的经历，增强了我们团队的凝聚力 |
| 27 | Relatedness | When my ideas for modifying AI outputs are recognized by teachers or classmates, I feel a sense of professional identity. | 当我修改AI方案的想法得到老师或同学认可时，我会有一种专业认同感 |
| 28 | Relatedness (R) | After using AI, my professional communication with teachers and classmates has actually decreased. | 使用AI之后，我与老师、同学的专业交流反而减少了（R） |
| 29 | Learning Motivation | Exploring AI applications in education has made me more interested in my professional studies. | 探索AI在教育中的应用，让我对专业学习更感兴趣了 |
| 30 | Learning Motivation | Mastering the ability to use AI in teaching strengthens my identity with the future teaching profession. | 掌握运用AI进行教学的能力，让我对未来从事教师职业更有认同感 |
| 31 | Learning Motivation | I am willing to actively spend time learning AI-related knowledge to enhance my ability to teach with AI. | 我愿意主动花时间学习AI相关知识，提升自己运用AI教学的能力 |
| 32 | Learning Motivation (R) | After using AI, my enthusiasm and initiative in professional learning have actually decreased. | 使用AI之后，我对专业学习的积极性和主动性反而下降了（R） |

Note: (R) indicates reverse-scored items. These items were reverse-coded prior to analysis (i.e., 6 minus original score) to ensure consistent directional scoring across all dimensions.

## **A2. Formal Study Questionnaire (N = 600)**

The formal survey was conducted from September to October 2025 with an independent sample of 600 pre-service teachers who did not participate in the pilot study or qualitative interviews. Based on pilot study results, an AI usage behavior module was added, and minor wording adjustments were made to enhance clarity. All items were measured on a 5-point Likert scale (1 = strongly disagree, 5 = strongly agree). Reverse-scored items (R) were transformed prior to analysis.

****Part I: Demographic Information and AI Usage Behaviors****

| ****Variable**** | ****Category**** | ****Options**** |
| --- | --- | --- |
| Gender | 性别 | □ Male 男 □ Female 女 |
| Grade Level | 年级 | □ First year 大一 □ Second year 大二 □ Third year 大三 □ Fourth year 大四 |
| Academic Discipline | 专业类型 | □ Humanities and Social Sciences 人文社科类师范 □ Mathematics and Natural Sciences 数理科学类师范 □ Arts and Physical Education 艺术体育类师范 □ Other (please specify) 其他师范（请注明） |
| Number of AI Tools Used | 使用AI工具种类 | □ 0 types 0种 □ 1-2 types 1-2种 □ 3-4 types 3-4种 □ 5 or more types 5种及以上 |
| Weekly Usage Frequency | 每周使用频率 | □ Never used 从未使用 □ 1-2 times per week 每周1-2次 □ 3-5 times per week 每周3-5次 □ Almost every day 几乎每天使用 |
| Primary Usage Scenario | 主要使用场景 | □ Lesson plan/teaching design 撰写教案/教学设计 □ Literature search/organization 查找/整理文献资料 □ Thesis writing 辅助毕业论文写作 □ Teaching materials/courseware 制作教学课件/微课 □ Other 其他______ |
| Primary Usage Mode | 主要使用方式 | □ Direct copy 直接复制使用 □ Minor modification 稍作修改后使用 □ Careful review and modification 认真审阅并修改后使用 □ Reference only 仅作为参考，主要靠自己完成 |

****Part II: AI Literacy and Psychological Needs Scale****

Items 1-32 are identical to those in the pilot study questionnaire (see A1 above). Based on pilot results, no substantive changes were made to the core scale items.

## **A3. Sample Independence Statement**

It is important to note that ****three independent samples**** were involved in this study:

| ****Sample**** | ****Purpose**** | ****N**** | ****Data Collection Period**** |
| --- | --- | --- | --- |
| Pilot Study Sample | Scale validation and refinement | 304 | April-May 2025 |
| Formal Survey Sample | Main quantitative analysis | 600 | September-October 2025 |
| Qualitative Interview Sample | Thematic analysis | 44 (36 students + 8 instructors) | November-December 2025 |

Participants in the pilot study did not participate in the formal survey or qualitative interviews. The qualitative interview sample was purposively selected from the formal survey respondents based on stratified sampling criteria.

**Dataset S1 | Pilot Study Data**

Sample size: N = 304 (independent sample, did not participate in the formal survey)

Items: 32 core items + 4 demographic variables

Reverse scoring: 8 reverse-scored items have been transformed (6 minus original score)

**Dataset S2 | Formal Survey Data**

Sample size: N = 600 (independent sample, did not participate in the pilot study or interviews)

Items: 32 core items + 4 AI usage behavior items + 4 demographic variables

Reverse scoring: Same as pilot study

The raw data for both datasets (after reverse scoring transformation) are provided.
